# High Fat Diet Causes Depletion of Intestinal Eosinophils Associated with Intestinal Permeability

**DOI:** 10.1371/journal.pone.0122195

**Published:** 2015-04-02

**Authors:** Andrew M. F. Johnson, Anne Costanzo, Melanie G. Gareau, Aaron M. Armando, Oswald Quehenberger, Julie M. Jameson, Jerrold M. Olefsky

**Affiliations:** 1 Department of Medicine, Division of Endocrinology and Metabolism, University of California San Diego, San Diego, California, United States of America; 2 Department of Pharmacology, University of California San Diego, San Diego, California, United States of America; 3 Department of Gastroenterology, University of California San Diego, San Diego, California, United States of America; 4 Department of Biology, California State University San Marcos, San Marcos, California, United States of America; College of Tropical Agriculture and Human Resources, University of Hawaii, UNITED STATES

## Abstract

The development of intestinal permeability and the penetration of microbial products are key factors associated with the onset of metabolic disease. However, the mechanisms underlying this remain unclear. Here we show that, unlike liver or adipose tissue, high fat diet (HFD)/obesity in mice does not cause monocyte/macrophage infiltration into the intestine or pro-inflammatory changes in gene expression. Rather HFD causes depletion of intestinal eosinophils associated with the onset of intestinal permeability. Intestinal eosinophil numbers were restored by returning HFD fed mice to normal chow and were unchanged in leptin-deficient (Ob/Ob) mice, indicating that eosinophil depletion is caused specifically by a high fat diet and not obesity *per se*. Analysis of different aspects of intestinal permeability in HFD fed and Ob/Ob mice shows an association between eosinophil depletion and ileal paracelullar permeability, as well as leakage of albumin into the feces, but not overall permeability to FITC dextran. These findings provide the first evidence that a high fat diet causes intestinal eosinophil depletion, rather than inflammation, which may contribute to defective barrier integrity and the onset of metabolic disease.

## Introduction

The penetration of bacteria or bacterial products, such as LPS, across the intestinal barrier is associated with the development of chronic inflammation and metabolic disease in both obese humans and mice [1,2,3,4,5,6]. Evidence suggests that reduction of tight junction protein expression and degeneration of epithelial barrier integrity is a feature of this permeability [4,7,8,9]. However, the underlying and associated mechanisms remain ill-defined.

The intestinal immune system is an effective immunological barrier to infection based on its juxtaposition to the extensive community of micro-organisms referred to as the intestinal microbiota [10]. Indeed, the intestinal immune system is often referred to as a “firewall” preventing the systemic penetration of bacteria. This barrier function has metabolic implications, since microbial signals in locations distinct from the gastrointestinal (GI) tract can trigger inflammatory processes, such as those which can cause insulin resistance. Indeed, intravenous infusion of low levels of endotoxin to mimic bacterial penetration is sufficient to render mice glucose intolerant [2].

It has previously been suggested that a high fat diet (HFD) or obesity results in pro-inflammatory changes in the ileum and proximal colon of rodents, contributing to intestinal permeability [11,12,13,14,15,16]. These changes include foci of NF-κB activation and 1.5–6 fold increases in the expression of TNF-α and IL-1β [13,15,16]. However, such increases in cytokine expression have not been observed in all published studies [16], and neither histological nor cellular analysis of the colon revealed significant pro-inflammatory responses [14]. Cellular analysis of the small intestine in HFD fed mice remains unreported.

Residing within the lamina propria, macrophages, dendritic cells (DCs) and eosinophils are predominate cell populations. Intestinal macrophage/DC populations regulate both tolerance to the microbiota and host defense against infection. Specifically, macrophage/DC IL-10 production and induction of regulatory T (Treg) cells can promote tolerance, whereas depletion of intestinal macrophages renders mice susceptible to bacterial penetration [17,18]. Although quite abundant, intestinal eosinophils have been less extensively studied. Eosinophilia can be a feature of specific inflammatory conditions in mice and humans, such as food allergies, eosinophilic gastroenteritis, allergic colitis and inflammatory bowel disease (IBD) [19,20,21]. In these conditions, recruitment and expansion of eosinophils is promoted by cytokines (e.g. IL-5) and chemokines (e.g. eotaxins) and they play an active role in disease progression [19,20,21]. However, tissue-resident eosinophils can also promote epithelial repair and barrier function during homeostasis [22,23,24]. We sought to determine whether the intestinal permeability observed on HFD feeding and obesity is associated with alterations in small intestinal macrophage/DC or eosinophil populations.

## Materials and Methods

### Mice

C57Bl6/J and Ob/Ob were purchased from Jackson labs and CX3CR1GFP/+ mice [25] were bred in-house and experiments approved by UCSD Institute Animal Care and Use Committee (IACUC). Mice were maintained on normal chow diet (Lab Diet 5001) on a 12h/12h light/dark cycle up to 8–12 weeks of age prior to transfer to a high fat diet containing 60%kcal fat (Research diets, New Brunswick, NJ, D12492) and analyzed at the indicated time points. In diet switch studies, mice were provided with HFD for four weeks and the returned to normal chow for a further 12 days. Upon analysis, mice were euthanized with 75mg/kg Pentobarbital (Schering-Plough, Millsoboro, DE) by i.p. injection and the diaphragm cut.

### Fecal albumin ELISA

Fecal pellets were collected prior to the application of a high fat diet (day 0) and on 3 proceeding days, snap frozen in liquid nitrogen and stored at -80°C. Pellets were resuspended at 10mg/ml in sterile phosphate buffered saline (PBS) and the concentration of albumin determined by ELISA in accordance with manufacturer’s instructions (Bethyl labs, Montgomery, TX, E90-134).

### *In vivo* FITC Dextran assay

Permeability was determined using a protocol previously described [4]. In brief mice were fasted for 6 hours (7am-1pm) and orally gavaged with 600mg/kg 4kDa FITC dextran (Sigma-Aldrich, St. Louis, MO) from a 125mg/ml solution. 1 hour later 120μl blood was collected, stored on ice in the dark and centrifuged at 12000xg for 3mins. Plasma was then diluted 1:2 in PBS and the concentration of FITC dextran determined by fluorescence spectroscopy (excitation at 485nm and emission at 535nm) relative to a linear standard curve made using diluted FITC dextran solutions in plasma from untreated mice.

### Flow cytometry of small intestinal lamina propria leukocytes

The small intestine was dissected, washed in cold PBS and mesenteric fat removed. Peyer’s patches were excised, the intestine cut longitudinally and contents removed by washing three times in fresh cold PBS. The intestine was blotted on paper towel, washed a final time in cold PBS, and cut into pieces approximately 1 cm in length. Pieces were incubated for 30 minutes in HBSS media (without Ca^2+^ or Mg^2+^) (Life Technologies, NY, 14175) containing 5% FCS, Penicillin-streptomycin, 5mMEDTA at 37°C with agitation at 200rpm. The supernatant containing removed epithelial layer was aspirated, fresh media applied and the process repeated. The intestine was shaken in three changes of HBSS media (with Calcium chloride, Magnesium chloride and Magnesium sulphate) (Life Technologies, NY, 14025) containing 5% FCS and the pieces incubated for 35 minutes in the same HBSS media containing 0.5mg/ml Collagenase type II (Sigma-Aldrich, St. Louis, MO) and 40μg/ml DNaseI (Sigma-Aldrich, St. Louis, MO). The cell suspension was filtered through 100μm and 70μm filters each washed with 5ml cold HBSS media containing 5mM EDTA and cells pelleted by centrifugation at 400xg for 5 minutes. The approximate yield was between 5–10 million viable cells per intestine. 2 x 10^6^ cells were then incubated with 1:1000 dilution of Live dead aqua (Invitrogen, NY) and 1:100 dilution of anti CD16/32 antibody (eBioscience, San Diego, CA, 93) for 30 minutes on ice in PBS, washed twice and incubated with the following antibody panel in PBS containing 2%FCS, 2mM EDTA: CD45 NC605 (30-F11), F4/80PECy7 (BM8) (from eBioscience, San Diego, CA), CD11b-FITC (M170), Siglec F-PE (E50-2440), CD11c-APC or CD11c-v450 (HL3) (from BD Biosciences, San Jose, CA) and MHCII APCCy7 (Biolegend, San Diego, CA, M5/114.15.2). Cells were fixed overnight in BD stabilizing fixative (BD Biosciences, San Jose, CA), washed twice and acquired on a BD Canto RUO flow cytometer. During analysis all gates were set relative to “fluorescence minus one” controls containing all fluorochromes minus the one of interest.

### Fluorescence microscopy

The small intestine was isolated, the distal third dissected and rinsed with PBS. Tissue was then opened longitudinally, rolled mucosa side outwards and embedded in O.C.T. compound (Tissue-Tek, Sakura Finetek USA, Torrance, CA). 10μm sections were cut using a Leica Cryostat (Buffalo Grove, IL) and fixed with 4% methanol-free formaldehyde (Sigma-Aldrich, St Louis, MO). Sections were immunostained with antibodies directed against CD11b (Biolegend, San Diego, CA), MHCII FITC (Biolegend, San Diego, CA) and Siglec F (BD Biosciences, San Jose, CA) and mounted with SlowFade Gold Antifade medium containing 4′-6-Diamidino-2-phenylindole (Invitrogen, Carlsbad, CA). Tunnel staining was conducted using TACS2 TgT-Fluor In Situ Apoptosis detection kit (Trevigen, Gaithersburg) according to manufacturer’s instructions with cytonin used for permeabilisation and cobalt cation in the labelling reaction. Images were digitally acquired (Zeiss AxioCam HRC, Thornwood, NY) and analyzed using Image J software (rsb.info.nih.gov/ij/). For cell quantification, villi length was determined using Image J software measure tool and the number of cells quantified per villi. Three individual of mice were used per group, with a minimum of 20 images acquired.

### Monocyte tracking studies

Peripheral blood mononuclear cells were isolated from the blood of wildtype donor mice and monocytes enriched using EasySep mouse monocyte enrichment kit (StemCell technologies, Vancouver, Canada). Monocytes were labelled with PKH26 (Sigma-Aldrich, St. Louis, MO, PKH26GL), washed twice in PBS and resuspended at 2.5 x 10^7^ cells per ml in PBS prior to injection. 200μl (5 x 10^6^ cells) were injected i.v. retro-orbitally. The proportions trafficking to the intestine were determined 3–5 days later by cell isolation and flow cytomtery as described above.

### RNA extraction and quantitative PCR

The small intestine was opened longitudinally, contents removed and mucosa scraped away from muscularis using a clean razor blade and snap frozen in liquid nitrogen. RNA was extracted using Trizol (Life Technologies, NY) according to manufacturer’s instructions. 2μg RNA was converted to cDNA using a High-capacity cDNA conversion kit (Life Technoliges, Applied Biosystems, NY). Gene expression was measured by Quatnitative PCR using iTAQ university SYBR green super mix (Bio-Rad, Hercules, CA). The following primer sets were used for SYBR green Quantitative RT-PCR (5’-3’): β-actin FWD GGTTCTTTGCAGCTCCTTCGT, β-actin REV ATATCGTCATCCATCGCGAAC, CD11b FWD TGTGAGCAGCACTGAGATCC, CD11b REV ATGAGAGCCAAGAGCACCAG, CD11c FWD ACACAGTGTGCTCCAGTATGA, CD11c REV GCCCAGGGATATGTTCACAGC, TNFα FWD CCAGACCCTCACACTGAGATC, TNFα REV, CACTTGGTGGTTTGCTACGAC, IL-1β FWD CTTGGGATCCACACTCTCCAG, IL-1β REV AAATACCTGTGGCCTTGGGC, MCP-1 FWD AGGTCCCTGTCATGCTTCTG, MCP-1 REV GCTGCTGGTGATCCTCTTGT.

### Eicosanoid analysis

The distal third of the small intestine (ileum) was weighed, supplemented with a cocktail consisting of 26 deuterated internal standards, homogenized with 1 ml 10% methanol per 5mg tissue on ice and briefly sonicated. Samples were then purified by solid phase extraction on Strata-X columns (Phenomenex, Torrance, CA), following the activation procedure provided by the distributor. Samples were eluted with 1 ml of 100% methanol, the eluent was dried under vacuum and dissolved in 50 µl of buffer A consisting of 60/40/0.02 water/acetonitril/acetic acid = 60/40/0.02 (v/v/v) and immediately used for analysis. Eicosanoids used for primary standards in standard curves as well as their deuterated analogs were from Cayman Chemicals (Ann Arbor, MI) and Biomol (Enzo Life Science, Framingdale, NY).

Eicosanoids were analyzed by reverse-phase liquid chromatography, using a 1.7uM 2.1x100 mm BEH Shield Column (Waters, Milford, MA) and an Acquity UPLC system (Waters, Milford, MA) and mass spectrometry, using an AB SCIEX 6500 QTrap mass spectrometer equipped with an IonDrive Turbo V source (AB SCIEX, Framingham, MA), as previously described. Eicosanoids were quantitated by the stable isotope dilution method using standard curves from the deuterated internal standards. To calculate the amount of eicosanoids in a sample, ratios of peak areas between endogenous eicosanoids and matching deuterated internal eicosanoids were calculated. Ratios were converted to absolute amounts by linear regression analysis of standard curves generated under identical conditions.

### Measurement of intestinal permeability in Ussing chambers

Segments of mid-jejunum and distal ileum were cut along the mesenteric border and mounted in Ussing chambers (Physiological instruments, San Diego, CA) exposing 0.09 cm^2^ of tissue area to 4 mL of circulating oxygenated Ringer’s buffer maintained at 37°C. The buffer consisted (in mM) of: 140 Na^+^, 5.2 K^+^, 1.2 Ca^2+^, 0.8 Mg^2+^, 120 Cl^−^, 25 HCO_3_^−^, 2.4 H_2_PO_4_^−^, 0.4 HPO_4_^2−^. Additionally, glucose (10 mM) was added to the serosal buffer as a source of energy, osmotically balanced by mannitol (10 mM) in the mucosal buffer. Agar–salt bridges were used to monitor the potential difference across the tissue and to inject the required short-circuit current (Isc) to maintain the potential difference at zero. This was registered by an automated voltage clamp and continuously recorded by computer. Conductance (G) was determined at baseline as an indicator of paracellular ion flux and expressed as mS/cm^2^. FITC labeled dextran (4 kDa, Sigma-Aldrich) was used as a probe to assess macromolecular permeability, and was added (2.2mg/mL final concentration) to the luminal buffer once equilibrium was reached. Serosal samples (200 uL) were taken at 30 min intervals for 2h and replaced with fresh buffer to maintain constant volume. The flux of FITC-dextran from the mucosa to the serosa was calculated as the average value of two consecutive stable flux periods (60–90 and 90–120 min) and expressed as pmol/cm^2^/h [26].

## Results

### High fat diet causes rapid onset of intestinal permeability

**Fig 1:**
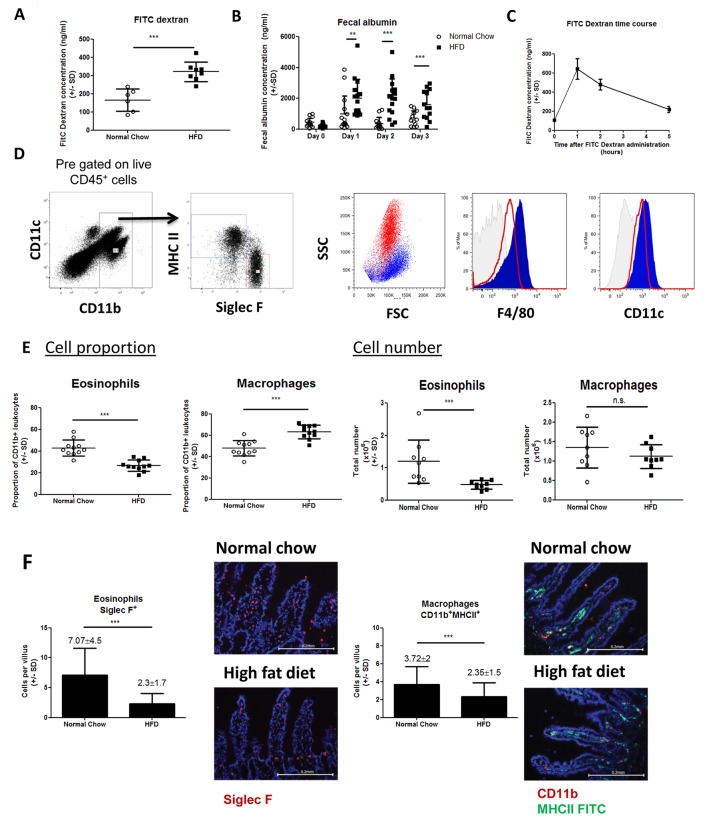
HFD causes intestinal permeability and eosinophil depletion. Intestinal permeability was determined after 7 days HFD by FITC dextran assay (A) or at day 0, 1, 2, 3 HFD by fecal albumin (B). Each data point represents an individual mouse from one (FITC dextran) or two experiments (fecal albumin). (C) Plasma FITC dextran concentration was measured at time 0, 1, 2 and 5 hours after oral administration to indicate the peak of permeability. Each data point is the mean (+/-SD) of 4–8 mice. (D) Gating strategy to identify eosinophils and macrophages. Lamina propria cells were pre-gated as live, CD45^+^. CD11b^+^ cells were compared for expression of Siglec F and MHC-II revealing two distinct populations. Siglec F^+^ MHC-II^-^ eosinophils (red) show high SSC, intermediate F4/80 and CD11c expression whilst Siglec F^-^ MHC-II^+^ macrophages (blue) show low SSC, high F4/80 and CD11c expression. (E) Proportions and total number of eosinophils and macrophages in normal chow and 7 days HFD mice. Each data point represents an individual mouse from two experiments. (F) The total number of Siglec F^+^ (red) eosinophils and CD11b^+^ (red) MHC-II (green) macrophages per villus shown by immunofluorescence microscopy. Each graph represents multiple villi from >30 sections from 3 mice per group. *p<0.05, **p<0.01, ***p<0.001 (Mann-Whitney U test).

Following oral administration, FITC dextran concentration was higher in mice fed HFD for 7 days compared to chow fed controls (Fig 1A), indicating increased intestinal permeability as previously described [1,2,7,15,27]. Intestinal permeability can also be determined by analyzing albumin concentration in the feces and we found increased fecal albumin concentrations as early as 1 day after HFD compared to control mice (Fig 1B). Thus the onset of intestinal permeability is a rapid event following ingestion of a HFD, preceding the onset of obesity. In addition, we conducted a time-course experiment in HFD fed mice and found that peak appearance of FITC dextran in the plasma occurred at 1–2 hours following oral FITC dextran administration (Fig 1C), consistent with upper gastrointestinal tract permeability. For this reason we largely focused on the small intestinal immune response in subsequent studies.

### High fat diet causes depletion of intestinal eosinophils

To determine whether intestinal permeability was associated with changes in intestinal immune cell populations, we used flow cytometry to examine the small intestinal lamina propria derived cells in HFD and chow fed mice. After pre-gating for live CD45^+^ CD11b^+^ cells, two leukocyte populations could be easily distinguished based on mutually exclusive expression of the eosinophil marker, Siglec F, and the antigen presenting molecule, MHC-II (Fig 1D). Siglec F^+^ MHC-II^-^ cells also displayed a high side scatter profile characteristic of eosinophils. By contrast Siglec F^-^ MHC-II^+^ cells showed lower side scatter and expressed high levels of F4/80 and CD11c indicators of macrophages/DCs in the lamina propria (Fig 1D). Interestingly, however, Siglec F^+^MHC-II^-^ eosinophils also expressed both F4/80 and CD11c demonstrating that commonly used “macrophage” markers are not specific enough to distinguish macrophages and DCs from eosinophils in the intestine. Thus, using this gating strategy we defined Siglec F^+^ MHC-II^-^ cells as eosinophils and Siglec F^-^ MHCII^+^ cells as macrophages/DCs.

After 1 week HFD there was a marked reduction in both the proportion and the absolute number of eosinophils in the lamina propria, whereas macrophages/DCs remained unchanged in number and so were proportionally increased (Fig 1E). To confirm these changes, independent of the collagenase digestion process required to isolate cells, we quantified eosinophils and macrophages/DCs by immunofluorescence microscopy. Using this method in 7 day HFD mice we found a 67% decrease in eosinophils (Siglec F^+^ cells) per villus with only a minor change in macrophages/DCs (CD11b^+^MHCII^+^ cells) (Fig 1F). These data provide the first evidence that HFD causes a deficiency within the intestinal immune system associated with the onset of intestinal permeability.

### High fat diet does not cause intestinal inflammation

**Fig 2:**
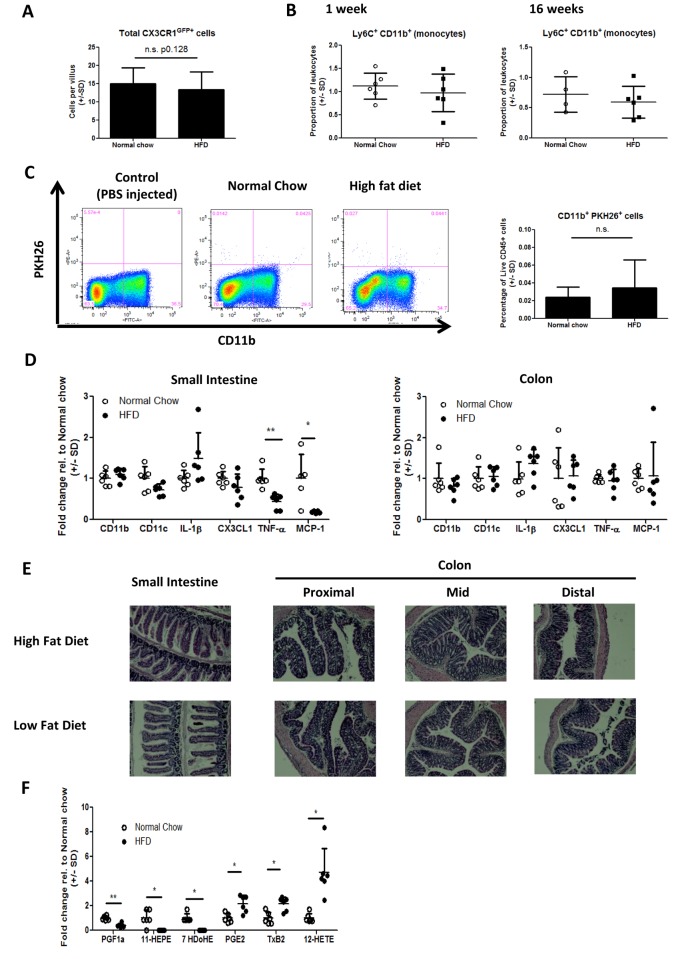
Lack of detectable inflammation in the intestine of HFD mice (A) CX3CR1^GFP/+^ monocytes/macrophages were quantified per villus by immunofluorescence microscopy. Multiple villi were assessed from >30 sections with 3 mice per group. (B) Lamina propria cells were isolated after 1 week or 16 weeks HFD and monocyte populations were analyzed as live, CD45+, CD11b^+^Ly6C^+^ cells. Each data point represents an individual mouse from one experiment. (C) The trafficking of adoptively transferred PKH26+ monocytes to the intestine is not enhanced in mice fed HFD for 8 weeks. Representative flow cyometry plots of a control receiving PBS in place of monocytes, normal chow recipient and HFD recipient mice are shown. Bar graph shows from n = 6 mice per group from one experiment. D) Inflammatory gene expression in the small intestinal mucosa of 1 week HFD or normal chow mice. *p<0.05, **p<0.01, ***p<0.001 (Mann-WhitneyU test). E) Representative H&E histology from the small intestine or colon of 6 week HFD or Low fat diet fed mice. F) Eicosanoid quantification in the ileum of mice fed HFD for one week or maintained on normal chow. Each data point represents an individual mouse from one experiment. *p<0.05, **p<0.01 (One-way ANOVA with Bonferroni post test).

Interestingly, unlike other tissues such as the liver or adipose [28,29,30], there was no evidence of inflammatory monocyte infiltrate or macrophage accumulation in the intestine of HFD mice. Specifically, using CX3CR1^GFP+^ mice, there was no increase in the number of GFP+ cells per villus in HFD mice (Fig 2A), nor was there an increase in lamina propria CD11b^+^ Ly6C^+^ monocytes (Fig 2B). We also measured *in vivo* monocyte tracking to the intestine by injecting fluorescently-labeled donor monocytes into HFD or chow fed mice and measuring their appearance in the lamina propria 5 days later. Only minimal migration of monocytes to the intestine could be detected (consistent with previously published monocyte tracking studies in leukocyte replete mice [31,32]) and there was no increase in the proportion of labelled monocytes in HFD mice (Fig 2C). Gene expression studies of small intestinal tissue showed no increased expression of CD11b, CD11c or IL-1β genes, but did reveal a decrease in the expression of TNF-α and MCP-1 genes in 7 day HFD mice compared to chow fed controls (Fig 2D). Analysis of the same genes in colon tissue revealed no significant changes in expression between 7 day HFD fed mice and chow fed controls (Fig 2D). Histological analysis of sections of small intestine and colon showed no evidence of pro-inflammatory changes, such as mononuclear cell infiltrate, epithelial hyperplasia or goblet cell depletion (Fig 2E). Lastly, analysis of eicosanoids present in the ileum indicated that PGF1α, 11-HEPE and 7-HDoHE were significantly reduced in HFD mice, whereas PGE2, TxB2 and 12-HETE were significantly increased (Fig 2F). Collectively, these data provides further evidence that, unlike the liver and adipose tissue, widespread inflammation is not a feature of the intestine after HFD feeding.

### Evidence for an eosinophil trafficking defect in HFD mice

**Fig 3:**
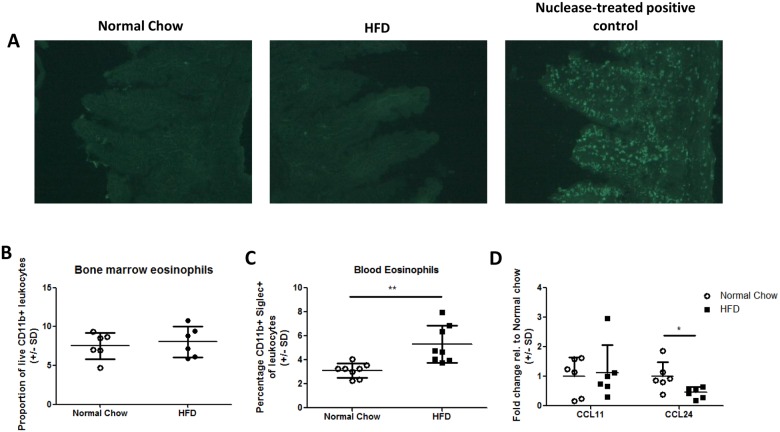
Evidence of an eosinophil trafficking defect in HFD mice. (A) Ileal sections from normal chow or 7 day HFD mice were stained for in situ apoptotic cells using TdT-Fluor staining protocol. Minimal apoptosis was detected in either condition relative to nuclease treated positive control. (B) Bone marrow and (C) peripheral blood leukocytes were isolated from 5 week HFD mice and proportions of eosinophils determined by flow cytometry. D) Expression of CCL11 and CCL24 genes in the small intestinal mucosa of 1 week HFD or normal chow mice. In all graphs each data point represents an individual mouse from one experiment. *p<0.05, **p<0.01, ***p<0.001 Mann-Whitney U test.

The HFD-induced intestinal eosinophil depletion could be due to eosinophil apoptosis or to defects in eosinophil development or migration. Tunnel staining of ileum sections from 7 day HFD or normal chow mice failed to indicate significant up-regulation of apoptosis in HFD fed conditions (Fig 3A). In addition there was no difference in the proportion of eosinophils detected in the bone marrow of HFD fed mice compared to normal chow controls (Fig 3B). Thus, it is unlikely that eosinophil apoptosis or defective eosinophil development are primary drivers of the eosinophil depletion upon HFD feeding. There was, however, a significant increase in blood eosinophils in HFD mice relative to chow fed controls (Fig 3C), which is consistent with there being a potential defect in eosinophil trafficking to the intestine. Indeed, HFD feeding led to decreased expression of the eosinophil chemoattractant, CCL24, but not CCL11, in the small intestine (Fig 3D). To test directly whether eosinophil trafficking was reduced in HFD fed mice, we measured the number of fluorescently labelled bone marrow-derived eosinophils in the intestine 3–4 days after intravenous injection. However, as has previously been reported using this method in naïve, unchallenged mice, we found minimal trafficking to the target tissue (<0.1percent of cells) of normal chow or HFD animals confounding definitive interpretation [33]

### Intestinal eosinophil depletion is caused by the fat content of the diet rather than obesity

**Fig 4:**
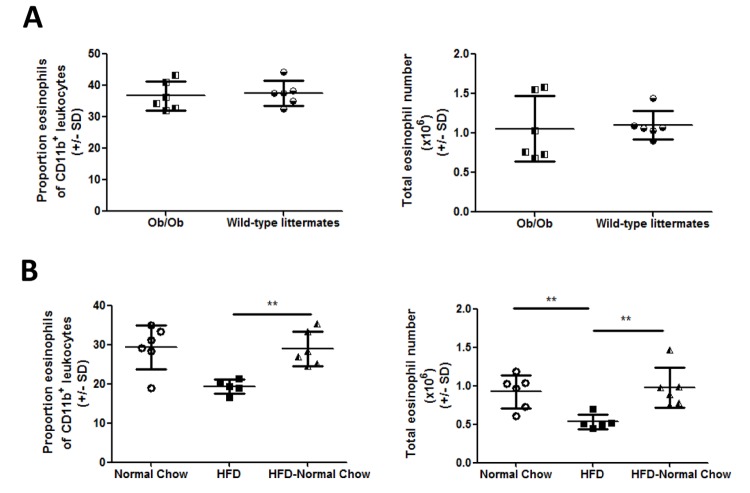
Intestinal eosinophil depletion is caused by HFD not obesity. (A) Lamina propria cells were isolated from 12 week old Ob/Ob mice and littermate controls or (B) HFD fed, normal chow fed or mice switched from HFD to normal chow (HFD-NC). Eosinophil populations assessed by flow cytometry as shown in Fig 1. Each data point represents an individual mouse from one experiment. *p<0.05, **p<0.01, ***p<0.001 Mann-Whitney U test.

To determine whether the high fat content of the diet or obesity itself was the cause of the eosinophil deficiency, we analyzed lamina propria eosinophils in Ob/Ob mice, since these mice become obese on chow diets. In contrast to the HFD mice, there was no detectable difference in eosinophil content in the intestine of Ob/Ob mice (Fig 4A). Furthermore, switching HFD mice back to normal chow for a period of 12 days completely restored eosinophils and macrophage/DCs to normal levels (Fig 4B). These results indicate that the eosinophil deficiency in HFD mice is related to the fat content of the diet.

### Comparison of intestinal permeability in HFD and Ob/Ob mice

**Fig 5:**
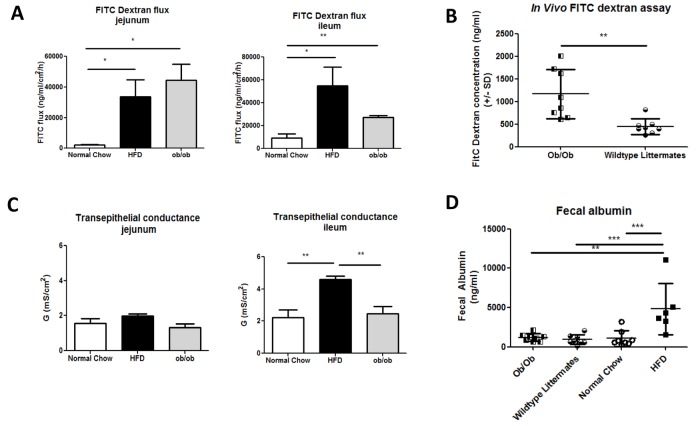
Intestinal permeability compared in HFD and Ob/Ob mice. (A) 4kDa Fitc-dextran flux across jejunum or ileum of normal chow (n = 4–6), HFD (n = 4) or Ob/Ob (n = 4) mice measured in Ussing chambers. (B) FITC-dextran concentration in the plasma of Ob/Ob mice and wild-type littermates following oral administration each data point represents an individual mouse (n = 8 per group) from one experiment. (C) Transepithelial conductance across jejunum or ileum of normal chow (n = 4–6), HFD (n = 4) or Ob/Ob (n = 4) mice measured in Ussing chambers D) Fecal albumin concentration in Ob/Ob mice, wildtype littermates, normal chow and HFD mice. Each data point represents and individual mouse from one experiment. *p<0.05, **p<0.01, ***p<0.001 by one way ANOVA (Ussing chamber analysis) or Mann-Whitney U test (*in vivo* FITC dextran or fecal albumin assays).

We also compared different aspects of intestinal permeability in HFD and Ob/Ob mice to determine whether eosinophil depletion was associated with a particular phenotype. Sections of proximal jejunum or distal ileum from chow fed, HFD fed or Ob/Ob mice were mounted in Ussing chambers. In this system, translocation of FITC dextran is a measure of total permeability (both transcellular and paracellular) while transepithelial conductance, is a measure of paracellular permeability [34]. Translocation of FITC dextran was increased in both the jejunum and ileum of HFD and Ob/Ob mice, indicating that intestinal permeability is increased in both models. Consistent with this, *in vivo* administration of FITC dextran to Ob/Ob mice showed a similar elevation in intestinal permeability as in HFD mice (Fig 5B). Interestingly, transepithelial conductance was specifically increased in the ileum of HFD fed, but not Ob/Ob mice and was not increased in the jejunum of either model (Fig 5C). Thus, ileal paracellular permeability is a feature caused by the HFD and is associated with eosinophil depletion. We did not detect the same increase in fecal albumin in Ob/Ob mice as was observed in HFD mice (Fig 5D), consistent with a HFD specific induction of paracellular permeability.

## Discussion

Obesity-associated chronic tissue inflammation is an important component of insulin resistance in obese subjects [35]. Recent studies have indicated that events arising in the gastrointestinal tract may play an etiologic role in driving systemic inflammation [1,2,4]. Thus, it has been well described that the gastrointestinal microbiota differ between the lean and obese state, and many studies have documented dysbiosis in obese rodents and humans [36,37]. This dysbiosis is associated with increased gut “leakiness” such that bacterial products, like LPS, and perhaps bacteria themselves, gain access to the systemic environment more readily in obesity [1,2]. Thus, it is hypothesised that signals derived from a leaky gastrointestinal tract promote systemic inflammation and insulin resistance in obesity.

Interestingly, unlike the liver or adipose tissue, we do not find a greater degree of inflammation in the small intestine or colon of HFD fed/obese mice compared to normal chow fed mice. Thus, we found no evidence for lamina propria macrophage accumulation in the small intestine, expression of standard inflammatory mRNA markers were either unchanged or reduced in the HFD small intestine and colon, and histologic analysis showed no evidence of tissue damage. Prior studies of the colonic response to a HFD showed similar data, with no immune cell accumulation or tissue damage with respect to cellular composition or histological analysis [14]. However, other studies have reported 1.5–6 fold increases in TNF-α expression in the proximal colon and ileum [12,13,15,16], and, using an NF-κB^eGFP^ reporter mouse, *Ding et al* showed foci of NF-κB activation along the length of the gastrointestinal tract following HFD feeding, which they concluded to be indicative of an inflammatory response [12]. One possible reason for this varying data could be the presence of varying microbiota offering different inflammatory potential. Indeed the inflammatory responses reported by *Ding et al*, were dependent on the presence of bacteria [12]. Alternatively, the whole tissue analyses presented here may not be sensitive enough to pick up highly localized regions of inflammation. Rather they give a broader of view of the immune status of the gastrointestinal tract.

The majority of studies on obesity-associated intestinal permeability have been conducted in rodent models, where increased permeability caused by HFD or obesity is reproducibly observed [1,2,5,8]. In man, one pilot study found no correlation between obesity and gut permeability [38], whereas, another found an association between small intestinal permeability and metabolic syndrome parameters, such as waist and abdominal circumference [6]. Interestingly, detection of 16s rDNA sequences in the circulation might be predictive of type II diabetes progression [3]. Here we show that intestinal permeability occurs rapidly after HFD feeding, preceding the onset of obesity. In addition, we show that, although overall permeability to FITC dextran can be observed in obese Ob/Ob mice in the absence of a HFD, specific measurements associated with paracellular permeability, such as albumin leakage into the feces and transepithelial conductance, are specific to the HFD fed condition.

One of the reasons for the difference in paracellular permeability between HFD fed and Ob/Ob mice could be the depletion of intestinal eosinophils, which was caused by HFD feeding, but not obesity alone. However, at present this remains an association as we have been unable to delineate a causal mechanism. Interestingly, a similar depletion of eosinophils also occurs in the adipose tissue following HFD feeding, and this coincides with polarization towards an M1-like macrophage phenotype and insulin resistance [39]. Eosinophils are highly granular leukocytes, generally found in small numbers (<5% of leukocytes) in the circulation and peripheral tissues [21]. The gastrointestinal tract is a notable exception to this, however, where tissue-resident eosinophils are reported to represent between 20–40% of leukocytes, a figure consistent with our data [40]. During inflammatory responses, such as allergy, eosinophils accumulate and produce pro-inflammatory and cytotoxic mediators with pathogenic consequences [19,20,21]. In the gastrointestinal tract this is true for a variety of conditions, such as allergic colitis, eosinophilic esophagitis and inflammatory bowel disease [19,20]. However, in the normophysiologic setting, eosinophils play a role in maintaining gastrointestinal homeostasis, including the maintenance of IgA-producing plasma cells, CD103+ dendritic cell and T cells, production and activation of TGF-β, and maintenance of the mucus layer [22,23]. Thus, we hypothesize that the depletion of intestinal eosinophils caused by a HFD could result in a state of nutritionally-induced relative immune deficiency and defective barrier function. Such an hypothesis is analogous to models of intestinal immune-deficiency caused by genetic deletion (e.g. TLR5-deficiency, ASC-deficiency) which result in penetration of bacterial products and the development of metabolic disease [41,42]. Further characterization of the intestinal immune system in HFD fed mice, the exposure of HFD fed mice to infectious challenge, and gain/loss of function experiments with eosinophil-deficient mice will be required to investigate this hypothesis fully.
